# Papillary Adenocarcinoma: A Rare Subtype of Lung Adenocarcinoma

**DOI:** 10.7759/cureus.44838

**Published:** 2023-09-07

**Authors:** Afaf Thouil, Abdelbassir Ramdani, Meriem Rhazari, Rachid Marouf, Hatim Kouismi

**Affiliations:** 1 Department of Respiratory Diseases, Research and Medical Sciences Laboratory, Faculty of Medicine and Pharmacy of Oujda, Mohammed VI University Hospital, Mohammed First University, Oujda, MAR; 2 Department of Surgical Oncology, Regional Oncology Center, Mohammed VI University Hospital, Oujda, MAR; 3 Department of Pulmonology, Mohammed VI University Hospital, Oujda, MAR; 4 Department of Thoracic and Cardio-Vascular Surgery, Faculty of Medicine and Pharmacy of Oujda, Mohammed VI University Hospital, Mohammed First University, Oujda, MAR

**Keywords:** smoking, small-cell lung carcinoma, lung, adenocarcinoma, papillary

## Abstract

Papillary adenocarcinoma (PA) of the lung is a specific form of lung cancer characterized by papillary structures in tumor cells. This type of cancer is relatively rare and has distinct pathological and radiological features that differentiate it from other types of lung adenocarcinomas. Determining the specific subtype of adenocarcinoma is a crucial factor in the choice of chemotherapy treatment. Detecting PA is fundamental, as it has both prognostic and therapeutic implications for patients with lung carcinoma. In this paper, we discuss two cases of young patients diagnosed with PA of the lung. The cases we present are particularly intriguing due to the relatively young age of the patients.

## Introduction

Papillary adenocarcinoma (PA) is a relatively rare subtype of lung adenocarcinoma in which papillary structures replace the underlying alveolar architecture [1,2]. The papillary structures must represent more than 75% of the tumor in a pathological study to confirm the diagnosis [1]. Determining the specific subtype of adenocarcinoma is a crucial factor in the choice of chemotherapy treatment. Detecting PA is fundamental, as it has both prognostic and therapeutic implications for patients with lung carcinoma.

## Case presentation

Case 1 

**Figure 1:**
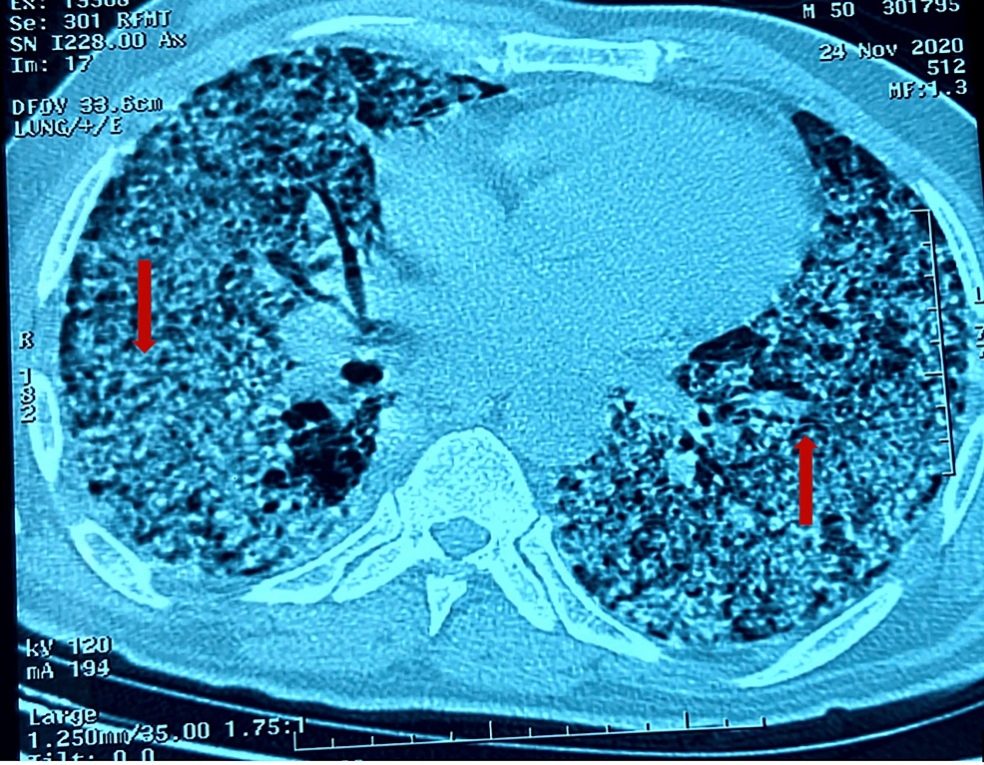
Thoracic computerized tomography showing micronodular lesions with extensive bilateral cavitation; some nodules are confluent in places occupying different lobes bilaterally (red arrows).

A 52-year-old male patient is a former chronic smoker. The patient had a history of pulmonary tuberculosis without any bacterial confirmation but had been receiving treatment for tuberculosis for the previous three months. The patient was admitted to the hospital due to worsening shortness of breath, fatigue, and confusion. During the clinical examination, the patient was restless and had low oxygen saturation levels (85%) while breathing room air. A thoracic computed tomography (CT) scan revealed the presence of small nodules and extensive cavities in both lungs, and some nodules are confluent in places occupying different lobes bilaterally (Figure 1).

The patient’s blood count was without particularity and revealed no hyperleukocytosis, hyper-eosinophilia, or lymphopenia. A bronchoscopy showed a strictly normal endoscopic aspect. Aspirations to search for acid-fast bacilli (AFB) were performed, as were investigations for *Aspergillus fumigatus*, *Pneumocystis jirovecii*, and tumor cells all came back negative. The Gene Xpert search of the fibro-aspiration fluid was negative. The search for AFB in post-bronchoscopy sputum also came back negative.

**Figure 2:**
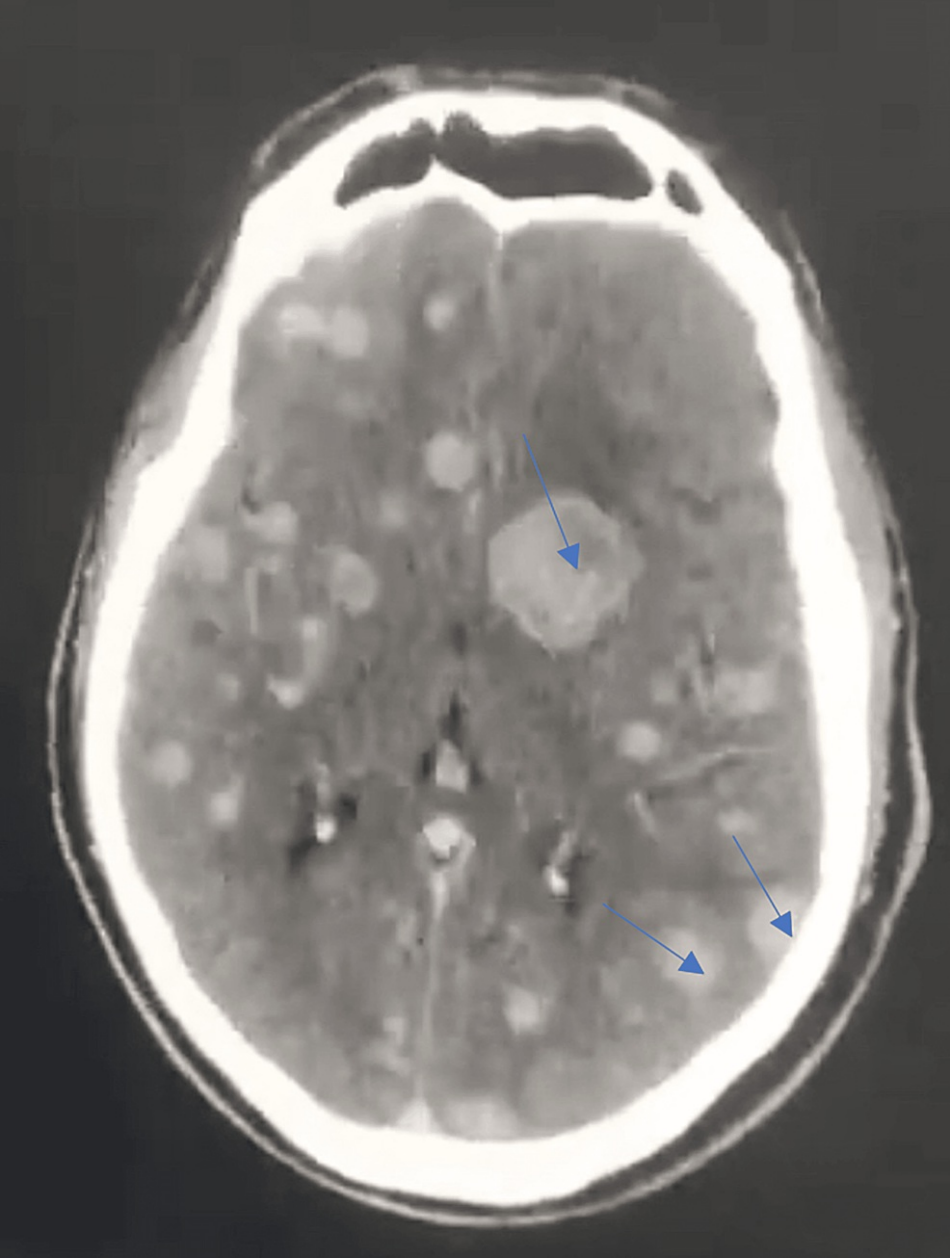
A brain CT scan revealed parenchymal nodular brain lesions without perilesional edema, resembling a balloon release (blue arrows).

Considering the patient’s neurological condition, a brain CT scan revealed parenchymal nodular brain lesions without perilesional edema, resembling a balloon release (Figure 2).

**Figure 3:**
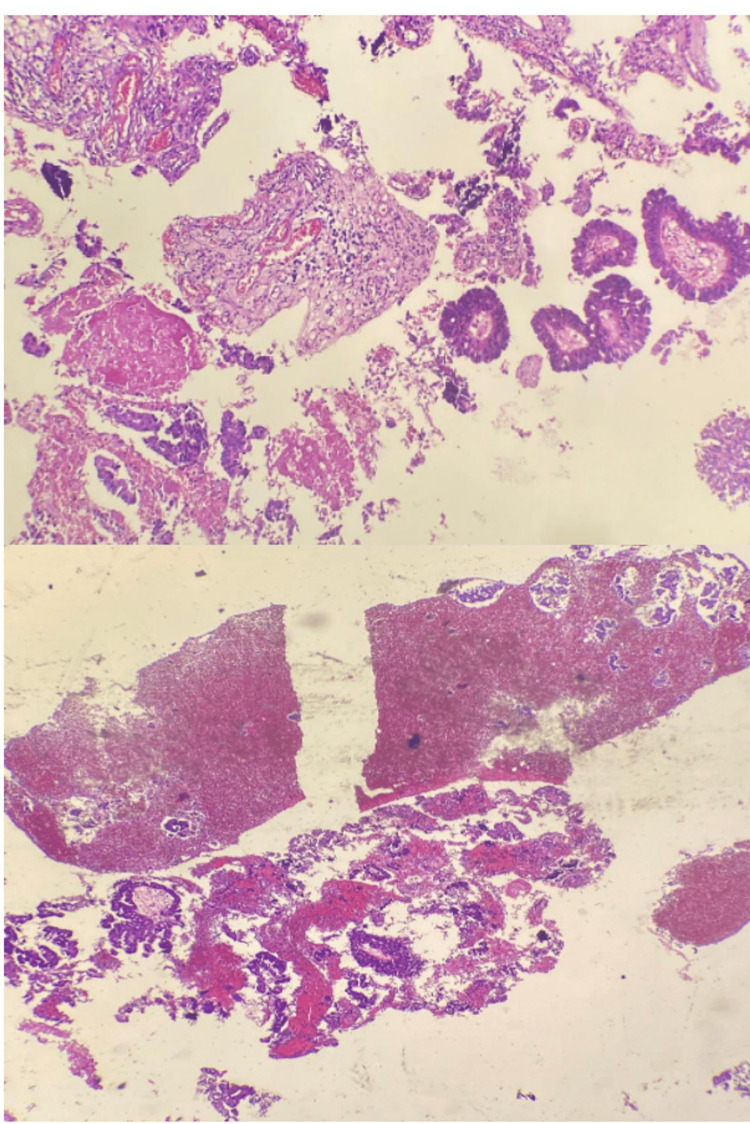
Pathological aspect showing well-differentiated carcinomatous proliferation of papillary appearance.

**Figure 4:**
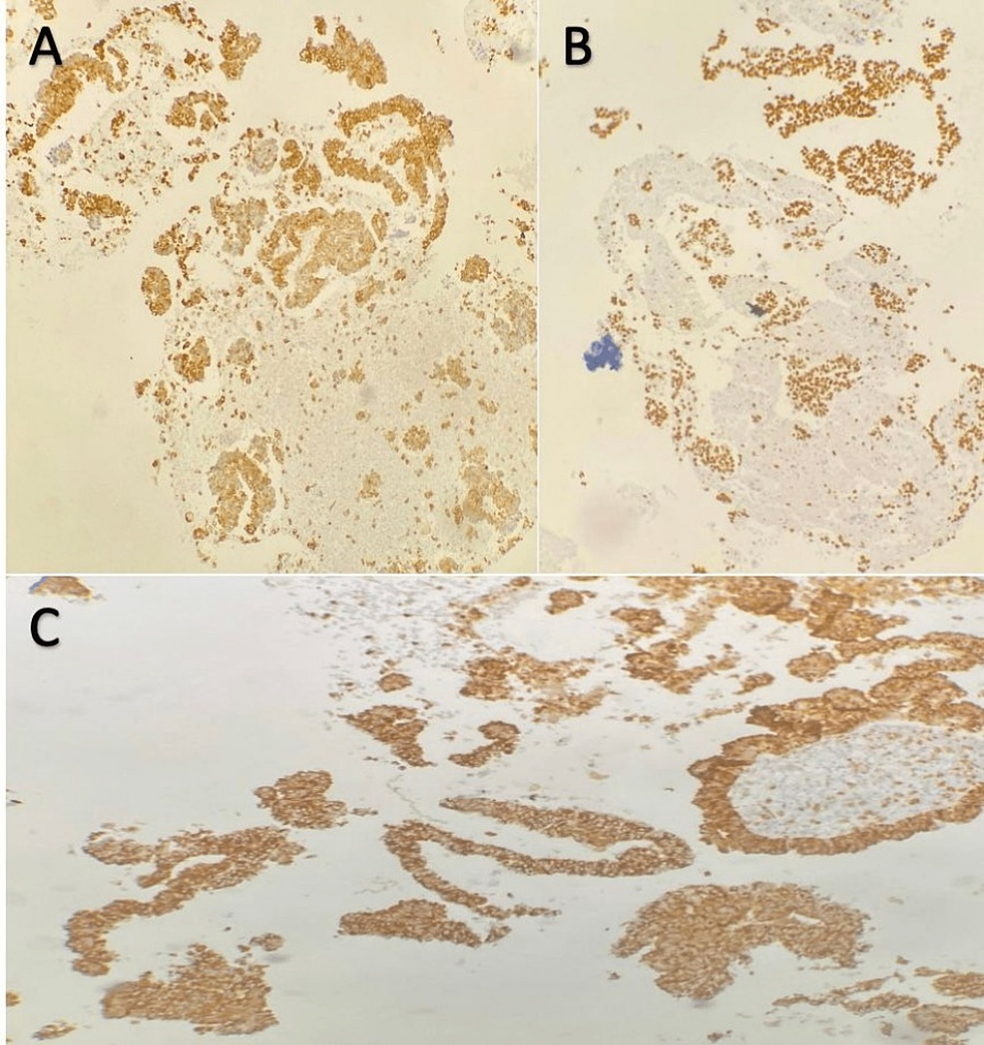
An immunohistochemical study: Positive marking of tumor cells by anti-CK7 antibody (a), anti-TTF1 antibody (b), and napsine A (c).

During a lumbar puncture, we noted an absence of cyto-bacteriological parameters for infection in the examined cerebrospinal fluid sample, and a prolonged culture of five days returned negative results. The protein and glucose levels in the cerebrospinal fluid were normal, and the Gene Xpert search was without particularity. The patient’s case was discussed in a multidisciplinary consultation meeting of the oncology department, which concluded with the indication for a stereotactic biopsy. The pathological study showed a well-differentiated carcinomatous proliferation of papillary appearance. This carcinomatous proliferation was made up of basophilic cylindrical cells. Their nuclei were round and finely nucleolated, showing discrete atypia and rare mitoses. The cells were arranged in papillae with a connective vascular axis, sometimes in anastomotic glandular tubes, in a fibrous and inflammatory stroma (Figure 3). The immunohistochemical study (Figure 4) showed positive results for anti-cytokeratin 7, anti-TTF1, and anti-napsin A antibodies, and negative results for anti-cytokeratin 20, leading to the conclusion of PA of bronchopulmonary origin.

An extension assessment did not reveal any bone, hepatic, or adrenal metastatic localization. The diagnosis of metastatic PA of bronchopulmonary origin at the cerebral level was made, and the patient was referred to radiotherapy and then to oncology for further management.

Case 2

**Figure 5:**
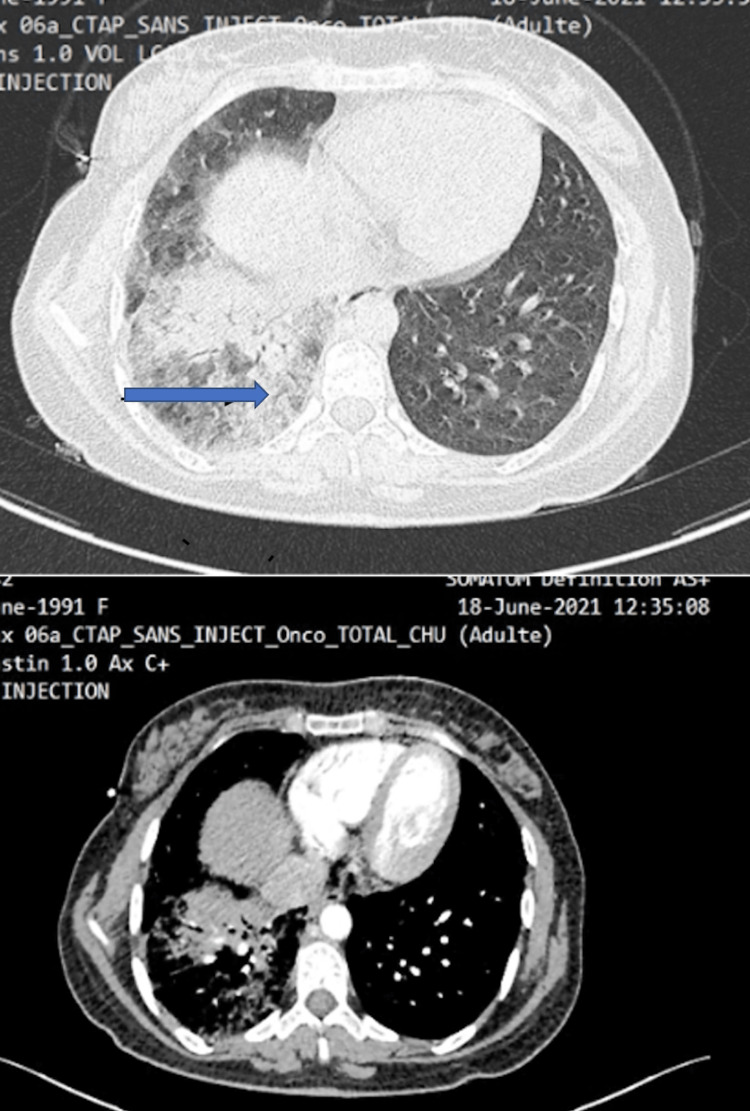
Thoracic CT scan revealed a focus of alveolar consolidation in the right lower lobe (blue arrow).

A 30-year-old non-smoking female patient, with a history of what appeared to be allergic rhinitis, was initially admitted for the management of exertional dyspnea associated with unquantified weight loss. Upon admission, the patient was conscious, with a stable heart rate and respiration and an oxygen saturation of 90% in room air. She showed pulmonary consolidation syndrome on the right side. Radiologically, a thoracic CT scan revealed a focus of alveolar consolidation in the right lower lobe (Figure 5).

As part of the etiological assessment, a hemogram was performed without particular findings, and a C-reactive protein test was negative, at 1.5 mg/l. The search for AFB in the sputum was negative. The Quantiferon test was also negative. Searches for *Aspergillus fumigatus* in the sputum and *Aspergillus *serology were both negative. The immunological assessment, including AAN and ANCA, was also negative. A bronchoscopy with bronchoalveolar lavage was performed without any particular findings. Tumor markers including CA 15-3, CA1 25, and CA 19-9 were all negative.

Given the negative results of the assessment, a trans-parietal biopsy was indicated. The histological study was in favor of a diagnosis of PA of the lung.

The extension assessment revealed a peripheral lower lobe pulmonary lesion with pleural extension and lymph node involvement without metastatic lesions. The case was discussed in a multidisciplinary meeting, and the decision made was to start neoadjuvant chemotherapy.

## Discussion

The pathological classification of lung adenocarcinoma encompasses various subtypes, including lipidic, acinar, papillary, micro-papillary, and solid with mucin production models. An accurate diagnosis and prognosis determination require definitive subtyping [1]. In this classification, PA is a rare subtype of lung adenocarcinoma [2,3]; due to its rarity, most PA studies consist of only a small series or single case reports. According to the World Health Organization, PA is characterized by massive papillary structures that replace the underlying alveolar structures of the lung [4]. This description applies to less than 10% of lung adenocarcinomas. PA is common in nonsmokers and is associated with invasive intrapulmonary metastasis, lymph node involvement, and poor overall prognosis [5]. While most patients diagnosed with this subtype are usually around the age of 65 [6], our two cases included patients younger than this.

Radiologically, PA often presents as an indistinct pulmonary nodule that may initially be mistaken for an atypical infection [7,8]. These malignant lesions are predominantly located in the upper lobes and are usually small in size. In particular, larger tumor sizes have been associated with more aggressive PAs [9]. The immunohistochemical features of this subtype have crucial prognostic implications [10]. Many studies have examined survival rates in aggressive lung adenocarcinoma, and the general consensus shows that patients with predominantly squamous adenocarcinoma have the best survival outcomes. This is followed by patients with acinar adenocarcinoma and PA. In contrast, patients with predominantly solid adenocarcinoma and mucus production tend to have poorer survival prognoses [9,11-13]. Surgical intervention, especially lobectomy or resection, has been proven as the optimal treatment and contributes to a better prognosis [14]. Notably, pneumonectomy leads to worse clinical outcomes, likely due to the significant loss of lung function [15]. The role of chemotherapy remains controversial [16].

## Conclusions

Papillary lung adenocarcinoma, a relatively rare form of primary lung tumor, presents a considerable challenge for medical practitioners, due to its difficulty to diagnose using standard cytological techniques. Surgical treatment is reserved for certain localized forms.
